# Scaling-up integrated type-2 diabetes and hypertension care in Cambodia: what are the barriers to health system performance?

**DOI:** 10.3389/fpubh.2023.1136520

**Published:** 2023-06-02

**Authors:** Savina Chham, Josefien Van Olmen, Wim Van Damme, Srean Chhim, Veerle Buffel, Edwin Wouters, Por Ir

**Affiliations:** ^1^National Institute of Public Health, Phnom Penh, Cambodia; ^2^Centre for Population, Family and Health, Department of Social Sciences, University of Antwerp, Antwerp, Belgium; ^3^Department of Family Medicine and Population Health (FAMPOP), Faculty of Medicine and Health Sciences, University of Antwerp, Antwerp, Belgium; ^4^Department of Public Health, Institute of Tropical Medicine, Vrije Universiteit Brussel, Brussels, Belgium; ^5^Department of Gerontology, Faculty of Medicine and Pharmacy, Vrije Universiteit Brussel, Brussels, Belgium; ^6^Centre for Health Systems Research and Development, University of the Free State, Bloemfontein, South Africa

**Keywords:** Cambodia, health system, scale-up, barriers, type-2 diabetes, hypertension, health system dynamic

## Abstract

**Background:**

Non-communicable diseases (NCDs) such as type-2 diabetes (T2D) and hypertension (HTN) pose a massive burden on health systems, especially in low- and middle-income countries. In Cambodia, to tackle this issue, the government and partners have introduced several limited interventions to ensure service availability. However, scaling-up these health system interventions is needed to ensure universal supply and access to NCDs care for Cambodians. This study aims to explore the macro-level barriers of the health system that have impeded the scaling-up of integrated T2D and HTN care in Cambodia.

**Methods:**

Using qualitative research design comprised an articulation between (i) semi-structured interviews (33 key informant interviews and 14 focus group discussions), (ii) a review of the National Strategic Plan and policy documents related to NCD/T2D/HTN care using qualitative document analysis, and (iii) direct field observation to gain an overview into health system factors. We used a health system dynamic framework to map macro-level barriers to the health system elements in thematic content analysis.

**Results:**

Scaling-up the T2D and HTN care was impeded by the major macro-level barriers of the health system including weak leadership and governance, resource constraints (dominantly financial resources), and poor arrangement of the current health service delivery. These were the result of the complex interaction of the health system elements including the absence of a roadmap as a strategic plan for the NCD approach in health service delivery, limited government investment in NCDs, lack of collaboration between key actors, limited competency of healthcare workers due to insufficient training and lack of supporting resources, mis-match the demand and supply of medicine, and absence of local data to generate evidence-based for the decision-making.

**Conclusion:**

The health system plays a vital role in responding to the disease burden through the implementation and scale-up of health system interventions. To respond to barriers across the entire health system and the inter-relatedness of each element, and to gear toward the outcome and goals of the health system for a (cost-)effective scale-up of integrated T2D and HTN care, key strategic priorities are: (1) Cultivating leadership and governance, (2) Revitalizing the health service delivery, (3) Addressing resource constraints, and (4) Renovating the social protection schemes.

## Background

The global burden of non-communicable diseases (NCDs) is rising, with a recent report showing that NCDs are responsible for 74% of deaths globally (1). Approximately one-third of NCD mortalities are related to cardiovascular diseases (CVDs) (2, 3). Around three-quarters of CVD-related deaths occur in low- and middle-income countries (LMICs) and more than half of these deaths result from hypertension (HTN) and diabetes (2, 4, 5).

The burden from these two diseases is large in LMICs: two-thirds of an estimated 1.28 billion adults aged 30–79 years with HTN, worldwide, and four-fifths of people with type-2 diabetes (T2D) live in LMICs (6, 7). The impact of this burden will hinder the health system's performance toward achieving the Sustainable Development Goal (SDG) targets for NCDs including target 3.4, which aims to “reduce premature NCD mortality by one-third” and target 3.8, which aims to “achieve Universal Health Coverage (UHC), which has implications for a wide range of NCD-related promotion, prevention and treatment interventions” (8). Evidently, the UHC service coverage index for NCDs in 2020 showed a slower gain at roughly 60% globally (9).

Similar to many other LMICs, T2D and HTN are two major NCDs that posed a burden for the Cambodian health system with a 3.3% prevalence of T2D and a 23.5% prevalence of HTN among adults aged 40–69 years in 2016 (10). However, in 2020, the prevalence of HTN was 35.2% among adults aged 40 years and older, while only one-third achieved a complete continuum of care and good health outcome (11).

In an attempt to tackle the issues of the health system to achieve the SDG targets for NCDs, numerous strategies targeting T2D and HTN have been introduced among LMICs, for example, development of the healthcare service organization including incentive-based, coordinated and organized teams; community-based interventions including raising awareness; multifaceted, policy interventions targeting integrated care and leadership; and multiple domain interventions (12, 13). In Cambodia, the government and partners have introduced a multitude of interventions to ensure service availability. These include the following interventions: (1) The establishment of *Non-Communicable Disease (NCD) clinics* at the referral hospitals (RHs), (2) The introduction of the *World Health Organization Package of Essential Non-Communicable Disease Interventions (WHO PEN)* program in health centers (HCs), and (3) The expansion and integration of the community-based *MoPoTsyo's Peer Educator Network* (11, 14).

Many initiatives designed to address the burden of T2D and HTN in LMICs have been around for years, yet there is little evidence of scaling-up those interventions to a larger population. In Cambodia, the above-mentioned interventions have been implemented for several years, yet they remain at small scales (only 31 NCD clinics for hospital-based care out of a total of 125 RHs (24.8%), 121 HCs for HC-based care out of a total of 1,221 HCs (9.9%) and 20 operational districts operated community-based care of MoPoTsyo out of a total of 102 operational districts (19.6%) in the whole country) and lack evidence of their scalability. To ensure universal supply and access to T2D and HTN care for Cambodians, these interventions need to be scaled-up to a wider population in different geographical settings.

According to the WHO, scaling-up means “deliberate efforts to increase the impact of successfully tested health interventions so as to benefit more people and to foster policy and program development on a lasting basis” (15). Over the past few years, the concept of scaling-up a health intervention has become popular due to which many initiatives have been undertaken to explore an effective small-scale intervention to be scaled-up (16–19). To accelerate this progress, understanding the gaps within the health system that slow down the process of having large-scale interventions for T2D and HTN is needed.

Despite the need for scale-up, the weakness of the health system—financial constraints, healthcare worker shortage, drug shortage, poor health information management systems, health service delivery challenges, and poor governance—may hinder the scale-up process, as has been studied in many LMICs (20–25). However, these weaknesses of the health system on T2D and HTN scaling-up have not yet been studied in Cambodia.

Therefore, an in-depth analysis of the current capacity and interrelatedness of each element of the Cambodian health system is required to identify levers for change and feed into the scale-up's strategic plan. For a successful scale-up, comprehensively understanding the health system into which the interventions are introduced and developing a clear strategic plan is crucial. The health system plays an important role in successful T2D and HTN management since these diseases require a comprehensive and cross-cutting approach to manage, for instance, life-long healthcare support, early case detection, psychosocial promotion, self-management, and medical support (26). To have a sustainable scaled-up intervention, it is essential to integrate disease-specific care programs for T2D and HTN into all system-wide functions of the health system “building-blocks” such as leadership and governance, financing, health workforce, health information systems, supply chains, and service delivery (27, 28). Achieving an effective approach for integration into the functions of the health system requires the active participation of the government and health system decision-makers in the process (29, 30). Our study focuses on the macro-level, meaning policy, political or structural context of a country, e.g., overall healthcare system, laws, and regulations, where results are used by government leaders and decision-makers to inform health policy regarding the state of healthcare services (31, 32). We aim to explore the macro-level barriers to the health system that have so far impeded the scaling-up of integrated T2D and HTN care in Cambodia by using a health system dynamic framework that focuses on linkages between different elements of the health system, stressing how outcomes and goals are achieved as a result of complex interactions of those elements (33).

The analysis will inform policy-makers about the barriers to health system performance, which can help to guide the strategic plan of scaling-up integrated T2D and HTN care in Cambodia.

## Methods

The design of this study was a qualitative method that comprises the articulation between (i) semi-structured interviews, (ii) a review of the National Strategic Plan and policy documents related to NCD/T2D/HTN care, and (iii) direct field observation for an in-depth exploration of the macro-level barriers. Qualitative research is the most appropriate method to explore complex phenomena such as health system dynamics. Qualitative methods include document analysis, observations of the researcher, and in-depth interviews, which convey the perception of respondents. The combination of these different sources allows a flexible approach to gathering information on implementation and how this is influenced by the health system context.

- Semi-structured interviews comprise 33 key informant interviews (KIIs) and 14 focus group discussions (FGDs). KIIs helped to explore the insight of key informants (KIs) at the national level who works on policy formulation, and KIs at the district level who oversight the implementation of current policies, implementation of T2D and HTN care and their perceived barriers to, and strategies of, scaling-up. FGDs were conducted to explore and understand the perception of front-line healthcare providers including community health workers (CHWs) regarding the existing barriers and gaps in the current implementation.
- To have better interaction during the interviews and gain insight into the current implementation and policy, a review of policy documents (standard operating procedures (SOPs), guidelines, strategic plans, etc.) using qualitative document analysis was done pre- and post-interviews. This was for interviewers to have a full understanding of the key themes or issues of the health system on T2D and HTN intervention mentioned in the documents, then compared it to the real practice at the health facilities. This method allows researchers to identify gaps between written guidelines and real implementation.
- A direct field observation at selected study sites was conducted to provide an in-depth overview, especially of the key related documents at the health facilities including patient records, registration forms, record of medicine stock, financial report of each source of revenue, and available database on the health financing and health information system since these two components were well-studied by complementing between interview and direct observation. Observations were recorded in a research field note.

### Selection of study participants and settings

The researchers reviewed the National Strategic Plan for the Prevention and Control of NCDs (2013–2020) (34) and identified the relevant KIs associated with interventions related to NCDs. After a list of KIs was compiled, senior researchers who specialized in the field of health policy and systems research were consulted to refine the list of potential KIs, which ended up with 18 potential KIs at the national level who are experts in different elements of the health system as well as chronic disease management. The participants are from diverse backgrounds including different departments of MoH whose roles are critical in each component of the health system building block, non-government organizations, health service providers at the national level and local level to ensure maximum variation of study participants.

Since the care initiative for T2D and HTN in Cambodia mostly took place at the primary care level, the relevant KIs at the provincial level were included, increasing the number to 33 potential KIs for the in-depth interviews (Appendix 1). In addition to the in-depth interviews, we also conducted FGDs with frontline healthcare professionals at the local level to identify their perceived barriers to, and gaps in, the current implementation.

The study was conducted at five purposively selected operational districts (ODs): OD Daunkeo, OD Kong Pisei, OD Sort Nikum, OD Samrong, and OD Pearaing. The selection of the five ODs is well-described in previous publications (11, 35). The study was conducted from June to September 2019.

### Data collection

The research protocol was approved by the National Ethics Committee for Health Research (No. 115/NECHR dated 29 April 2019) in Cambodia.

Two different interview guides were used for semi-structured interviews: one for the KI interviews (Appendix 2) and another one for the FGDs (Appendix 3). The main topic of the KI interview guide was the perspective on the current care for T2D and HTN, policy, scale-up, and recommended strategies, while the topic for the FGD interview guide was the current implementation of T2D and HTN care, perceived facilitators, and barriers to scaling-up and proposed solutions.

Two researchers conducted KI interviews (SVC asked the question and NSL/HVN took notes) and three researchers conducted the FGDs (SVC was a moderator, the NSL/HVN was an observer, and NSL/HVN took notes). Each interview was conducted in the local language and lasted 50–60 min.

To ensure rigor in data collection, data triangulation is used to enhance the trustworthiness of the information provided and to produce a more comprehensive overview of the T2D and HTN care, and participant selection is diverse in different areas of the health system in relation to chronic disease management to enable the research question to be addressed appropriately.

Participants were well-informed about the objectives of the study, voluntarily consented to an interview, and signed a consent form before being interviewed. All KI interviews were held at their respective offices and FGDs were held away from the respective health facilities to avoid influence from other healthcare workers. All interviews during which recording was allowed were transcribed by research assistants familiar with qualitative methods; a few interviews in which recording was not permitted were written in an interview note format. Confidentiality of the recordings and transcripts was maintained by the research team throughout the data collection, analysis, and publication.

We followed an iterative cycle to determine the data saturation which the data analysis occurred concurrently with data collection. The research team simultaneously conducted interviews and SVC developed the coding. When no new themes were being emerged from the interview, the team agreed that data saturation has been achieved.

### Analytical framework

**Figure 1:**
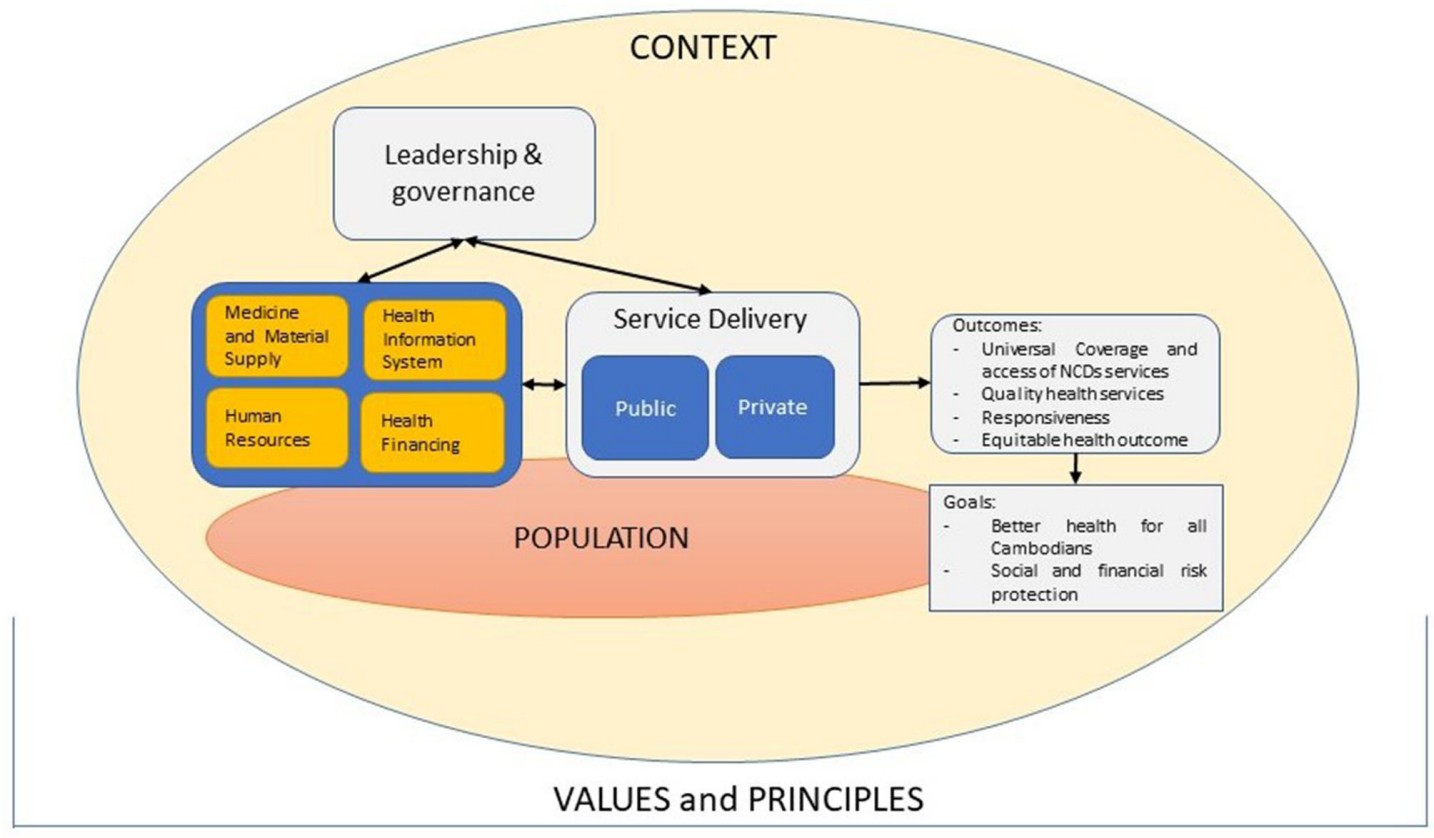
Adapted health system dynamic framework for scaling-up integrated T2D and HTN care in Cambodia.

The health system dynamic framework developed by van Olmen et al. (33) was adapted to analyze the macro-level barriers to the health system for scaling-up T2D and HTN care in Cambodia (Figure 1). The framework identifies ten health system components: (1) Values and principles, (2) Goals and outcome, (3) The context, (4) Leadership and governance, (5) Service delivery, (6–9). Organization of resources (human resources; financing; infrastructure and supplies; knowledge and information), and (10) The population.

This framework was used to map barriers to the health system elements in thematic content analysis. All of the interviews were read by the first author (SVC) to immerse herself in the data.

All codes and sub-codes that emerged during the analysis were grouped in a thematic framework using NVivo software (NVivo qualitative data analysis software; QSR International Pty Ltd.). Discussions were carried out with the research team to have a final code tree and definition of each sub-theme as illustrated in Table 1. We used the framework to emphasize how the health system should be geared toward outcomes and goals that are based on the explicit choices of values and principles of the Cambodian health system. Therefore, element (1) Values and principles, (2) Goals and outcome, (3) The context, and (4) The population are illustrated in this section.

**Table 1:** Code tree and definition.

**Theme and sub-theme**	**Definition**
**1. Leadership and governance**	
1.1. Policy Guidance	Strategic vision including technical knowledge and information, political and negotiation skill to steer the process of achieving goals and outcomes of the health system.
1.2. Accountability	Responsiveness and obligation of the governance to deliver the health services of the health system.
1.3. Coordination and collaboration	Coordination and collaboration between different important actors to balance delivery of public health services for target population and achieving goals of the health system.
**2. Health financing**	
2.1. Mechanism of funding	Major source of health financing for health sector and for public health facility.
2.2. Resource allocation	Funding on the health services to steer the delivery of T2D and HTN care.
**3. Human resources**	
3.1. Competence of healthcare workers	Limited capacity of the local health care providers on T2D and HTN care.
3.2. Availability of healthcare workers	Insufficient number of human resources for health.
**4. Medical supply**	
4.1. Poor supply and availability	Lack of medicine supply to the health facilities, number of supplies did not meet the shortfall in medicine and less variety of essential medicines to be used in each health facility.
4.2. Poor Medicine Request Procedure	Lack of evidence-based for medicine request.
**5. Health information system**	
5.1. Data availability	The limited availability of local data base to generate concrete evidence on T2D and HTN for policymakers.
5.2. Fragmentation of data source	The separated functional and purpose of each health database in the country.
**6. Health service delivery**	
6.1. Health services and delivery platform	Different characteristic and channels of T2D and HTN health services delivery.
6.2. Providers of health services	Characteristic of health service provider for T2D and HTN.
6.3. Acceptability of provided health services	Quality of health services provided as perceived by population.

#### Values and principles

The overall value-based commitment of the Cambodian Ministry of Health (MoH) is “Rights to Health for all Cambodians and Equity”.

Following these overall values and principles of the health system, the National Strategic Plan for the Prevention and Control of NCDs (2013–2020) shared a vision where “non-communicable diseases are effectively and equitably prevented and controlled for people in Cambodia, and the burden of non-communicable diseases on households and society is minimized” (34) and set the four principles of selection priority strategies for action related to NCDs, which are: 1. Priorities based on the burden of diseases, 2. Step-wise approach based on feasibility, 3. Prioritize cost-effective and equitable interventions, and 4. A whole government response.

#### Goals and outcome

The goals stated in the Cambodian Health Strategic Plan 2016–2020 are “improved health outcome of the population and increased financial risk protection” meaning to ensure access to, and coverage of, high-quality health services and financial access to quality health services by all Cambodians. This implies the universal coverage and access to quality NCD-health-services and responsiveness of the health system toward the population's needs and country context.

#### Context

Cambodia is going through a demographic transition. The Cambodian population is increasingly urban; the general population census reported that the urban population as a percentage of the total population increased from 19.5% in 2008 to 39.4% in 2019 (36). The report also indicated that the aging trend of the Cambodian population, with an increase in the older adult population aged 60 years old and above from 6.3% in 2008 to 8.9% in 2019, implies more need for treatment of NCDs and long-term healthcare services (36). The health system is facing a growing epidemic of NCDs that shared 69% of the causes of mortality in Cambodia in 2019 (37). The rising burden (prevalence and mortality), aging population, and urbanization pose challenges to the structure and delivery of the health system. Therefore, to tackle this rising burden of NCDs, especially T2D and HTN, there is a need to strengthen, modify, and expand the existing structure of the health system in different ways.

#### Population

Cambodian's most dominant healthcare provider is private care. The first provider sought among household members who needed care for illness, injury, or other health problems in the last 30 days was highest for private care, which accounted for around 70% in 2016 and 2019 (38, 39). Furthermore, a majority (59%) of people with chronic diseases were diagnosed and treated in private facilities (40).

For the Cambodian population to access care, there were some barriers reported in a previous study such as physical barriers, financial barriers, quality of care, knowledge of users, and socio-cultural barriers (41).

## Results

### Leadership and governance

#### Policy guidance

An important sub-theme that emerged from the interviews was the complexity of what and how to scale-up. The respondents believed that a roadmap is important to help inform the development of the new strategic plan, which in turn provides effective implementation. Even with the plan to update the National Strategic Plan for the Prevention and Control of NCDs (2013–2020), it is still not a comprehensive guideline for implementation. Some respondents agreed that the roadmap designed specifically for T2D and HTN will be more content-specific than the strategic plan for the whole NCDs approach. It can guide the important steps that should be taken to achieve the goals and outcomes of the health system.

“*The achievements of the current National Strategic Plan have not reached the target yet due to the lack of human resources, funding, and database. We might need a document, so-called ROADMAP, as a guiding document on what we should start from for the scale-up.” - Representative from policy-makers*

“*We can learn from the Maternal and Child Health program on how their progress is remarkable… they have a fast-track initiative as their roadmap with which they know what to do, how, when and their source of funding to do each step of their intervention. It is clear and achievable.” - Representative from implementers*

#### Accountability

Some respondents raised their concern about the influence of donor funding on the health policy process or set up national health agenda. When the proportion of external funding is greater than government investment in health, setting the priorities of the health policy process might be affected by their control of financial resources. The donor's ability to control indirect financial and political incentives might decrease the MoH's accountability and ownership because MoH's obligation to be answerable to the national health agenda is shifted by the donor health agenda. Respondents pointed out the necessity of local evidence from in-country research to set the priorities in health that could increase the MoH's or government body's accountability to the population's need in health service delivery and health agenda setting for the country's health goals.

“*Role of stewardship from the ministry or government is very important. While donors or development partners provide financial assistance, plans should be designed and led by the local people (government officials at the ministry) who are well-known of the context.” - Representative from policy-makers*

“*If the government sees more prevalence and evidence based on the burden, they need to scale-up. The demand and supply should be analyzed. I think (enabler) government support (or political support) is crucial. Every intervention always has challenges and for the scale-up, I think we lack resources (funding and human resources). The finding on the scale-up itself will be evidence-based, before the decision on scaling-up.” - Representative from policy-makers*

Another key point raised was the lack of willingness and commitment from the front-line health providers. For an implementation to be successful, it requires their leadership with high commitment and willingness to invest. These two points determine the pursuit of efficiency in health policy implementation and health service delivery at the local level. Respondents were concerned that healthcare managers did not understand their roles as a leader to take the lead with their own commitment and to not rely on the lead of non-government organizations (NGOs).

“*For an OD or RH or HC to implement the activity, it requires willingness and motivation. They depend too much on [name of NGO], so they are not willing to do it on their own. They do not start working hard for themselves to generate more user-fee, but just want a commission from [name of NGO].” - Representative from policy-makers*

“*I see that the supply cannot be your big barrier when the HC manager has a high willingness to implement health services. There is a HC at which I admired their manager the most for pooling staff all together to just work for the HCs and they can earn a very high income to share among the staff. However, this is a special case… you rarely find a leader like him; we cannot make other HCs to be accountable when they themselves do not have a commitment to do so.” - Representative from policy-makers*

#### Coordination and collaboration

Multi-sectoral collaboration between different important actors for health services such as line ministries, external partners, and non-government organizations is vital for large-scale implementation; however, respondents thought each actor did not understand this. All the initial implementations of each organization should be complementary in order to alleviate the potential of health intervention. If they focus only on their own mechanisms and do not stress the importance of collaboration for mutual benefits, Cambodia might fail to have a scaling-up approach nationwide.

“*Scaling-up T2D and HTN intervention requires a multi-actor approach and we need to open the space for their involvement as a complementary approach. We cannot do it alone and in a separate way.” - Representative from NGOs*

“*I like the phrase ‘triangle to move the mountain', which I learnt at the conference. To me, policy-makers, technical experts and the target population are the essential three angles to move the mountain to achieve health for all. Therefore, everyone is needed in the process to work together, deal together to move the mountain, but we need to ensure we have a strong coordinator to keep everyone entertained in the process. If not, they leave.” - Representative from policy-makers*

### Health financing

#### Mechanism of funding

There are three major health financing sources in Cambodia: (1) The government's general revenues, (2) Donors' development assistance, and (3) Individual out-of-pocket payments (OOP) for receiving the services. The government's general revenues are spent on compensation for employees, pharmaceuticals, materials and services, and consumption of fixed capital.

The current source of donors' development assistance on NCDs is from a multi-donor trust pooled with government financing called the Health Equity and Quality Improvement Project (H-EQIP) which supports increasing the number of ODs enabled (met all the supply-side conditions) to provide quality HTN and T2D screening and treatment services with an amount of USD 1.5 million for a period of 3 years (2018–2021).

Respondents were also concerned that this only source of funding for NCDs is not enough for large-scale effective implementation.

“*The leaders always support us in every activity and program as long as we can guarantee the funding from external donors. The government budget is not enough to do the work alone. However, external donors cannot guarantee long-term work either.” - Representative from policy-makers*

Based on the National Health Account (42), NCDs continued to account for around 21% of total health spending, which is relatively low compared to the budget for Maternal and Child Health (MCH) or infectious diseases. OOP remains the top contributor to health spending (around 60% in 2016), which was also the case for NCDs.

“*There is an imbalance between total health expenditure for NCDs and HIV/AIDS. As you see, there are high mortality rates for NCDs, but they receive a very small proportion of health expenditure. While HIV/AIDS is reducing its mortality rate, it is still receiving high health expenditure. It sounds ridiculous to me and this should be considered seriously. However, I think a voice from NCDs area needs to be heard by top policy-makers or the government leader through sharing economic impact, economic gain such as investment case with them. They need to be convinced through such things.” - Representative from policy-makers*

The main funding streams to public health facilities level are (1) Government budget, (2) Service delivery grant (fixed lump sum grants and performance-based grants), (3) User-fee, and (4) Social health protection schemes (42).

Regarding serviced delivery grants, fixed lump sum grants are funded entirely by the Royal Government of Cambodia and allocated in fixed amounts to HCs and RHs for operational expenditures in addition to the operational budgets defined in the annual operation plan. Performance-based grant refers to the grant disbursed to health facilities by the quarterly scoring of the quality of services under H-EQIP that can be used for both operational expenditures and staff incentives (with the formula of 20%:80% of the total grant).

User-fee is collected from the non-poor population for services used at HCs and RHs. The revenue from user-fees is allocated as 60% for staff incentives, 39% for health facility management, and 1% to be transferred to the treasury every month.

Respondents raised the fact that the current user-fee charges for public health facilities are disproportionate to the required treatment of T2D and HTN. The treatment interval should preferably be increased to 1 month or 3 months; however, the current rate of user-fee is unstandardized and low, which would not be adequate for the increased treatment interval for T2D and HTN.

“*The way how the fee charges are different and unstandardized at each health facility might be a factor in different performance of NCD services. We were asked to charge an out-patient fee, which is around 4,000 to 5,000 riels [USD 1.0 to USD 1.25] for health centers and 10,000 to 15,000 riels [USD 2.5 to USD 3.75] for referral hospitals. This rate defines the duration of medication given to patients. Lack of medicine supply is already a problem at each health facility.” - FGD implementer*

This funding stream remains a challenge, especially in response to frequent shortfalls in the regular medicine supply system. Even with percentages allocated from the service delivery grant or user-fee to operational expenditure, respondents complained about how the operational expenditure largely covers the medicines purchased to compensate for the shortfall of regular supply and delays the expenditure on another need of facility development.

“*We were told that fixed lump sum grant and user-fee are for us to purchase the medicines to supply the short-fall, but it did not happen just once; it is almost every month that we need to use this grant to purchase medicines. Do you know how much user-fee is collected at each health facility? It is not much and we plan to use it to develop another section of the facility too. It is not a healthy way for us to keep using this compensation style. What if there is a way to keep the grant for another line of operational budget and have at least a moderate enough supply of medicine?” - FGD implementer*

In Cambodia, there are two types of social health protection schemes: the Health Equity Fund (HEF) and the National Social Security Fund (NSSF). Among the total Cambodian population (~16 million), 38.5% had been covered by these two schemes by 2019 (43). The HEF is a financing scheme that enables poor people to access and utilize healthcare services free-of-charge in public health facilities including NCD-specific service packages and those service costs are borne by the Royal Government of Cambodia (44). The HEF covered 2,471,241 members from poor households in 2019 and there were a total of 1,311 health facilities that operated the HEF among 1,474 public health facilities (43). However, this scheme covers only the households identified as “poor” for utilizing services at public health facilities, while a majority of Cambodian people (60%) with chronic diseases were diagnosed and treated in private facilities (40) and the highest utilization was from the 1–9 years and the 20–35 years age groups (45).

The NSSF is designed for salaried employees, civil-servants, ex-civil servants, and veterans. NSSF healthcare scheme was introduced in 2016 and had a total of 1,698,759 members by the end of 2019 (43). The NSSF coverage is still low compared to the total population and still leaves those from the informal sector or non-registered workers behind.

Many respondents were not satisfied with the NSSF's reimbursement rate and complained about the delay in reimbursement that leaves the health facilities stuck in getting the money to purchase medicines/supplies or to compensate for previous expenses.

“*I am happy when more people use the NSSF card, but my concern is their reimbursement rate for T2D and HTN. We get the reimbursement rate as out-patient consultation, which is around 5,000 riels (USD 1.25). That's why we can give them the medicine for 1 week because this rate cannot compensate the amount of medicine we offer to the patients. We always ask the NSSF to revise the rates to at least give us a better compensation as the HEF does.” - Representative from implementers*

In addition, the reimbursement rate can only offer patients a medicine supply for a short period of around 5–7 days according to the regular out-patient consultation fees. This short period of medicine supply increases the number of patients visiting the health facilities for a follow-up leading to treatment and a higher chance of loss to follow-up due to distance, travel costs, and no companion to the health facility visits. Respondents suggested having an innovative benefits package to encourage their continuum of care. If this approach is adopted by the social protection scheme, it even encourages more people to access and adhere to care.

“*We have applied the NSSF reimbursement policy in our facility, but the problem we are facing now is they take longer than 3 months to reimburse our money back. In the period of 3 months without money coming back in as compensation for the medicines we provided to those patients with NSSF cards, we are unable to re-supply the medicines.” - FGD implementer*

“*For chronic disease patients, we need a reasonable fixed price to charge them. This disease requires frequent treatment, which increases the patients' spending. So, we need an innovative package of social protection to cover them as a continuum of care.” - Representative from policy-makers*

#### Resource allocation

Respondents raised the common challenge of the direction of donor funding, in which the implementation of certain interventions is based on the availability of external funding. Donors' or partners' intervention often comes with their own purposes and the pre-defined objectives and indicators that seem essential for their investment only. Since the government's investment is low in NCDs, the goal of implementation is always following what the donor wants to see.

“*When the donor's target or preference changed, adaptation was also made accordingly. For example, [name of NGOs] used to prioritize support to care for type-2 diabetes and hypertension through the WHO PEN at health centers, but now their focus has been shifted toward type-1 diabetes with support to 10 hospitals. In order to continue the support for type-2 diabetes among adults, the department had to find a way to adjust the budget (of NGOs) so that some amount could be left for it.” - Representative from policy-makers*

### Human resources

#### Competence of healthcare workers

The team of researchers assessed the training curriculum provided by the Department of Preventive Medicine and observed that there is an absence of leadership and management training for key staff. Apart from technical training, it is crucial to train key staff and integrate the leadership spirit into their daily work to boost their accountability.

“*Capacity for management and leadership is limited. The Ministry of Health should be strengthening their capacity. The training should be conducted at the PHD [Provincial Health Department] or OD levels (build core team) wherein they can provide continuous training to their health centers later on.” - Representative from the National Hospital*

Training is conducted only once at the beginning of the implementation and there is an absence of refresher training. Respondents demanded refresher training annually to strengthen the skill of trained healthcare providers and to equip new staff to be able to provide services when there is a change of staff. With the same level of knowledge, they can shift tasks or rotate schedules, which keeps the services running continuously.

“*I cannot guarantee that all of the staff stay at the same place and were sent to the training. They tend to change after the probation period, which I cannot control, especially when only two people are invited for the training. I think the MoH should provide more space for us in the training and conduct refresher training to ensure that we are updated every year.” - Representative from HCs*.

#### Availability of healthcare workers

Scaling-up, argued respondents, requires mutual-interest and investment in the place where the intervention is taking place. However, some HC staff focus on their own business and have left the facilities behind. Some staff only apply what they learn to their private practices.

“*As long as they do not benefit much from public health facilities compared to their own private practice, there is no way they are willing to initiate or take care of the intervention that is implemented at their HC.” - Representative from implementers*

Another cross-cutting challenge was the number of healthcare staff that is lacking in all areas of the public health facilities due to being under-incentivized. Limited qualified health human resources for NCDs often hinder the speed of scaling-up. Even if health human resources are well-trained, the lack of supporting resources poses a challenge to their performance and tendency to stay longer at public health facilities.

“*Additionally, doctors and nurses who are working at the HC level are not trained sufficiently in providing care and treatment to patients with diabetes and hypertension. Peer educator networks are only active in a minority of areas. Existing HCs have very limited capacity in diagnosing and treating type-2 diabetes and hypertension.” - Text in the SOP for T2D and HTN treatment in primary healthcare*

### Medical supply

#### Poor supply and availability

Another huge concern was the limited availability of medicines at the HC level, which are not complementary to the patient's conditions. This could lead to the worst-case scenario of complications arising by using medicines that are not suitable for their health conditions.

“*Talking about the HCs' medicine availability, it is not as many as compared to the RH. At the RH, we have many choices for HTN or T2D medicine depending on their [patients'] condition. I am afraid when scaling-up allows HCs for follow-up treatment, what can the HC do about it? They have a lack of medicine even for general use, so if scale-up requires them to have a different group of medicine, this is even truly impossible for HCs.” - Representative from implementers*

Supply in materials used for the intervention is equally important to medicines. Respondents raised the fact that materials for the NCD intervention are supplied only at the beginning of the implementation. They suggested having some materials being supplied by the central medical store, so they do not have to use the user-fees (which are usually not so high for HCs) to expend on materials. This continuous supply will help HCs to have at least something they can rely on for further implementation.

“*The strips are provided at only the beginning; HCs need to use their own money to purchase. I think a plan needs to be arranged, so it will be a part of materials can be supplied from the central medical store. Machines are supplied without maintenance checks, so if they break, HCs cannot find the repairer or the maintenance fees exceed their capacity to pay.” - Representative from NGOs*

#### Poor medicine request procedure

A related barrier identified in the study is the procedure for medicine requests which is lack in providing evidence-based reports to support the request. Data on patient care is needed to prove the amount of medicine requested/needed in each health facility. Some medicines are high in stock due to a lower number of cases to be treated, not because of enough supply. Other medicines are short in stock because there were a greater number of cases to be treated. Therefore, evidence is needed for the right estimation and right distribution of medicines.

“*One problem is that for a period of treatment, we cannot identify the exact amount of T2D medicine needed because the medicine is prescribed differently for different conditions and the method of administration (some use once per day, some use two pills per day and some use twice per day with HTN medicine).” - Representative from policy-makers*

### Health information system

#### Data availability

The researchers have identified that there is no centralized, updated, and well-structured data source for NCDs in Cambodia. First, the NCD database is available at the NCD clinic, but it is not updated due to it being time consuming and the lack of personnel for data entry. Health Management Information System (HMIS) is available for all public health facilities, but the indicators for diabetes cannot differentiate between type-2 and type-1 diabetes. Data on diabetes and HTN are reported as cumulative cases, meaning, one cannot identify whether those cases are the same or different patients.

The STEPs survey has been conducted only every 5 years to capture risk factors for NCDs in Cambodia, and that limits the frequent update on prevalence and related factors.

The researchers identified the plan of the HEART CARE (software) for HCs with the WHO PEN in some HCs of Cambodia; however, respondents complained about the difficulties in getting it into real practice due to a lack of human resources, poor internet services, and the fact that it is not a web-based system.

“*The HEART CARE software plans to use tablets for data entry. Firstly, it will be done by the patients themselves, then the doctors, nurses and pharmacists. The last step is printed out at the pharmacy. We think that it is not practical because tablet use is complicated and it might get lost easily.” - Representative from policy-makers*

#### Fragmentation of data sources

For nation-wide implementation, the dataset for NCDs should be integrated and be able to be combined as a pooled database, which is convenient to capture the real performance of NCD services. However, the currently available database is often funded by different donors and serves different objectives. This leads to difficulty in getting those databases combined as one due to different indicators being used and the fact that the previously-designed platform was not intended for integration.

“*We used to have a meeting in March on combining PMRS [Patient Management and Registration System] with HEART CARE, but it seems impossible as we know that databases in Cambodia mostly are project-based. If we want to change the PMRS format, we have to call all around 200 HCs who are currently using the PMRS to learn about the update. It seems difficult and challenging, that's why we will pilot HEART as a stand-alone record first.” - Representative from NGOs*

### Health service delivery

#### Health services and delivery platforms

The public healthcare intervention for T2D and HTN has been concentrated at the primary care level.

The delivery platform between the NCD clinic and the WHO PEN are connected as a complementary function to each other. However, the coverage of this health intervention is limited with 31 NCD clinics of hospital-based care out of a total of 125 RHs (24.8% coverage), 121 HCs of HC-based care out of a total of 1,221 HCs (10% coverage) within 23 ODs of a total of 102 ODs in the whole country (22.5% coverage). The respondents stressed the importance of ensuring large coverage for universal supply.

“*If you compare the number of HCs with WHO PEN to the total number of HCs in the whole country, it is too small and how are we supposed to get everyone screened? We have it for years already, but the expanding progress is still low due to financial constraints. I understand that it should be time to scale-up.” - Representative from implementers*

Respondents raised the absence of monitoring and supervision from all levels concerning NCD implementation; therefore, they as implementers have never received feedback on their implementation.

The supervision and monitoring activity is suggested to be a crucial component as it helps guide the roads of implementation. By having this component, they are able to understand challenges, correct problems and help the program achieve its goal successfully.

“*The role of [department of MoH] in supervision and monitoring is important. It allows them to understand the challenge of the real implementation, but we never saw them coming to supervise or monitor”. - Representative from implementers*

“*The challenges for the WHO PEN are no evaluation and monitoring system, insufficient supply of essential medicines and no tracking of referral system.” - Representative from NGOs*

The poor referral system was raised as a barrier to ensuring a continuum of healthcare services for those patients who need a confirmatory diagnosis at the RH. The SOP for T2D and HTN management provides guidance on referring suspected cases to the RH; however, there is no guidance on how the referral system should be set up to refer people.

Most patients were from rural areas and have financial difficulties, making it difficult for them to go for further referral care and for the HC to track if the patient is referred successfully to the RH.

“*Currently, there are problems with referral of patients from the HC to the RH wherein we cannot track if they actually arrived at the referral hospitals after being referred.” - Representative from policy-makers*

#### Providers of health services

Cambodia has a mixed health delivery system of both public and private health providers. The low utilization of public health services remains a concern and over-utilization of the private health services might cause a higher proportion of OOP spending that could lead to catastrophic health expenditure. Therefore, respondents mentioned that the quality of public healthcare services should be improved by minimizing resource constraints.

Respondents explained the difficulties in gaining trust from people in their area of coverage due to their perceived lower service quality compared to private facilities. Quality of services depends on cross-cutting issues, which could be a lack of supply, competent staff, or materials (primarily as a result of resource constraints). This inhibits public health facilities from achieving their potential in providing high quality services.

“*Quality of service needs to be strengthened to gain users' trust. People want faster service, reasonable price and quality of care to be trusted. Even if it has a long waiting time but good quality, they will still come to the HC or the RH.” - FGD community health worker*

“*The quality of health service should be strengthened and trust in the community and patients should be built. This in turn will be a facilitator in our health system context.” - Representative from the National Hospital*

#### Acceptability of the provided health services

There was a mismatch between real implementation practice and the written SOP for T2D and HTN management (28). Many specific sections are written in the SOP, yet the implementation failed to reach the expectation due to different cross-cutting factors of the health service delivery. For instance, the CHW's function is written on the SOP as a component of implementation, yet the involvement (of CHWs) is low and almost absent due to the lack of incentives and capacity training.

Human and financial resources are the linked constraints to the quality of health services. Participants mentioned their hesitance in receiving public health services due to a lack of trust, long waiting hours, and inflexibility of appointment times compared to private health providers.

In light of the limited number of qualified public health providers, respondents discussed the importance of community support's function in NCD intervention. They raised the lesson learned from other vertical programs such as the malaria or MCH programs, which partly came from the active participation of CHWs in community out-reach, providing referral services, health education, and social support. However, issues remain on how they could engage, what we can offer them in terms of technical and financial support, and where do they belong in the model of T2D and HTN care.

“*Strengthening the role of the current village health support group (a group of CHWs for HC) is crucial. The (referring) problem is not only for T2D but also HIV. I recommend using the existing structure, even if it's harder and works slowly, but it will be sustained.” - Representative from policy-makers*

“*CHWs are crucial for a chronic disease intervention. All of the national programs in Cambodia employed CHWs to support their intervention and it was a success. NCDs should learn from them and start engaging CHWs.” - Representative from NGOs*

## Discussion

Our study is the first to apply the health system dynamic framework to assess each element and the inter-relatedness of the Cambodian health system for scaling-up a healthcare intervention. The framework was useful for an in-depth investigation of the barriers from each element of the system and the linkage across elements that hinder the scaling-up of integrated T2D and HTN healthcare services.

Cambodia is experiencing rapid urbanization and population aging which increases the need for NCD services. This emerging problem of T2D and HTN among other NCDs poses a challenge for the health system and, in reverse, the health system's functions determine the progress of this health intervention.

The major gaps in the health system that impede the scaling-up identified in our study are weak leadership and governance, resource constraints (dominantly financial resources), and poor arrangement of the current health service delivery. They were not found as stand-alone barriers, but cross-cutting barriers that need to be tackled as a whole system approach.

Although there is a growing recognition of NCDs (particularly T2D and HTN) as one of the prioritized areas in the health strategic plan 2016–2020, weak governance appeared to lag the performance of the intervention. In recognition of these two diseases' burden, the MoH has published the SOP (28) and the National Strategic Plan (34) for NCDs; however, the progress of getting those written strategic actions into real implementation is slow and remains dominantly decided by external partners. Previous studies conducted in Ghana, India, and Ethiopia also identified poor leadership and governance as a health system challenge for HTN and related NCD care (24, 46, 47). Similar to many LMICs, Cambodia's health system is tackling with competing priorities of diseases as well as non-disease sectors. In the framework of the SDG agenda, effective strategies by strengthening the capacity of the local government to increase accountability on the implementation of quality NCD service are essential. A previous study suggested that short-term leadership training had a positive impact on health personnel development as well as their organization in LMICs (48). Governance can be strengthened through the bottom-up approach of accountability between health service users and providers, or the top-down approach of accountability by holding policy-makers more accountable for services and their position influences the quality and coverage of services (49). In Cambodia, the government issued sub-decree number 193 on 4 December 2019 on the assignment of the health management function and health service delivery to the capital and province administration (50). This aims to bring accountability to the local level and bring health services closer to the population. However, this reform is still at an early stage of implementation and its progress has not yet been evaluated. Well-designed and implemented decentralization policy offers a variety of benefits to the health system especially maximizing the potential of the health system governance for NCDs (51).

The current state of governance on T2D and HTN is reflected in the government's financial commitment to NCDs. Even with the existence of a strategic action plan, resource constraints (finance and health human resources) limit the implementation. Financial resources play an important role for scaling-up in LMICs as indicated in a previous study (52). In another study, it has been shown that most LMICs have an underdeveloped health system with insufficient public spending and inadequate health insurance coverage (53). In addition to financial funding, financial protection strategy is found to be critical to ensure universal coverage. As indicated in our study, the coverage of the social protection scheme is low and the reimbursement fee for chronic disease management is unsatisfactory for the service providers. Therefore, a re-assessment of the user-fee and reimbursement fee for health services should be conducted to balance the services provided, medicines supplied, and required duration of follow-up for chronic disease management such as T2D and HTN. Evidence from LMICs has shown that insurance predicted a lower rate of people falling into catastrophic health expenditure and insurance coverage serves as a critical tool in promoting NCD healthcare (54).

Progress toward universal coverage of quality NCD service is suffering from a lack of investments in the health system. The disproportionate gap between the financial supply and the need for T2D and HTN might slow down the country's progress toward universal coverage of quality care. Therefore, the government needs to find an innovative funding mechanism through boosted tax revenue for NCDs. A case study from Tonga has shown that the NCD tax policy is used to support health goals and to help the country achieve universal coverage of NCD services (55).

The primary care level in Cambodia, particularly HCs and RHs, has prioritized MCH programs, so the addition of T2D and HTN services has been met with a lack of human resources and limited infrastructure to offer these services. Facilities were not previously designed to provide healthcare services for T2D and HTN, and that affects the arrangement of health service delivery especially for screening and health education. The poor design of the referral arrangement impedes the implementation of a comprehensive WHO PEN at the primary care level. It is plausible that together with the lack of staff availability and lack of competent healthcare workers (due to insufficient skills in T2D and HTN management), the quality of health services may be affected, hindering the community to trust the services at public health facilities. This requires a revitalization of the current version of health service delivery before setting a scale-up strategic plan. To revitalize the services, some strategies should be considered to ensure continuity of care by using health information technology to track the referral status of patients. This strategy has been used in the case of tuberculosis referral (56) and for NCDs in Sub-Saharan Africa (57).

Barriers related to the lack of human resources have been also identified in a previous study in Ethiopia (47). In light of limited health human resources, task-shifting to CHWs has been recommended as a potential strategy for T2D and HTN health service delivery (58, 59).

The shortfall in medicine supply has been a concern for Cambodia's health system “for ages” as mentioned by respondents. A limited supply of essential medicine was also imposed as a barrier to primary care services for cardiovascular diseases in Vietnam (60). Effective procurement of essential medicines and inclusion of more medicines into benefit packages for social protection schemes has been suggested to improve access to medicines for NCD care (61). In Cambodia, the social security scheme on healthcare for chronic diseases covered only those medicines stated “available” for respective public health facilities in the essential drug list. For those medicine out of the essential drug list or stated as “non-available” or “special need” for some health facilities, patients must pay themselves (62). A variety of medicines is needed for differing diagnoses of T2D and HTN, some of which may not be indicated as being available in the essential drug list for respective health facilities. If the NSSF does not cover the variety of medicines used for T2D and HTN, patients will have increased OOP and this impedes the health system from achieving its outcome. The reimbursement policy for a comprehensive package of essential medicines used for chronic diseases should be considered by expanding the list of reimbursable drugs.

Despite the functional HMIS, NCD indicators captured in the system are limited. A well-developed data management platform is essential for the country to generate reliable morbidity and mortality data for evidence-based policy development. The STEPs survey conducted routinely every 5 years is important; however, the long spacing interval of years between surveys is a concern. To respond to the poor-structured HMIS for NCDs in the short-term, another large survey conducted every 2 years to provide recent progress of NCD services in the country is needed to inform the experts through a national health progress report or scientific publication which could be used as input for potential data-driven policymaking. To track the progress of achieving its desired outcomes and goals, NCD-related indicators for T2D and HTN should be integrated into the health system monitoring framework.

To accelerate health system performance toward achieving the SDG agenda for NCDs especially target 3.4, many interventions need to be prioritized for Cambodia. A study on the efficient pathway and strategic investments to accelerate the progress toward SDG target 3.4 for LMICs has outlined many cost-effective interventions, yet each country needs to define their most urgent priorities plans for NCDs based on their local context such as the collection of local data to produce reliable estimates to local health planner for Malawi, and revision of health benefits package for a wide range of NCD intervention for Ethiopia (63).

While our study contributed an in-depth insight into the challenges of scaling-up an integrated T2D and HTN care in Cambodia, there are some limitations that must be noted. One potential limitation is the gender and type of health providers' imbalance in our study sample. A majority of the participants identified as KIs were male and came from public health facilities; however, they were the resourceful personnel who could provide us with clear insight into each element of the health system for T2D and HTN care leading to well-addressed challenges in the study. Our intervention of interest, to be assessed for the potential of scaling-up, is to be implemented at the primary care level of the public health sector, which can explain the dominance of respondents from the public sector.

## Conclusion

The health system plays a vital role in responding to the disease burden through the implementation and scale-up of health system interventions. Barriers across the entire health system need to be addressed for a (cost-)effective scale-up. Governance plays a central role in the decision to scale-up integrated T2D and HTN together with resource constraints and poor health service delivery. A clear strategic plan is essential by clearly designing a roadmap that provides guidance on how to scale-up, roles and responsibilities, and resource mobilization to implement each strategic action. For a sustainable scale-up, resources mobilized from government spending are needed.

For the health system to respond effectively to the local need and its context, and to gear toward the outcome and goals of T2D and HTN care services, key strategic priorities are: 1. Cultivating strong political leadership and governance with the focus on resource mobilization, 2. Revitalizing the health service delivery for NCDs for an effective future scaling-up of quality NCD care service, 3. Addressing resource constraints by inventing innovative strategies such as task-delegating to CHWs for some light functions and short-term surveys for updated data on NCDs, and 4. Expanding or renovating the social protection scheme.

Understanding these challenges is a key step in providing evidence to policy-makers on areas to be prioritized in Cambodia for effectively scaling-up integrated T2D and HTN care.

## Data availability statement

The datasets generated and/or analysed in this study are available from the first author upon reasonable request.

## Ethics statement

The studies involving human participants were reviewed and approved by the National Ethics Committee for Health Research. The patients/participants provided their written informed consent to participate in this study.

## Author contributions

SVC, JVO, WVD, EW, VB, and PI conceptualized the manuscript. JVO, WVD, and EW contributed to the application of the analytical framework. SVC analyzed the data and wrote the manuscript. PI contributed to the discussion section of the manuscript. All authors read, edited, and approved the final manuscript.
