# Supplementary material for: Scaling-up integrated type-2 diabetes and hypertension care in Cambodia: what are the barriers to health system performance?
Source: Front Public Health. 2023 Jun 2;11:1136520. doi: 10.3389/fpubh.2023.1136520 (PMC10272385; doi:10.3389/fpubh.2023.1136520)
Supplement: Supplementary file 1 [file Table_1.DOCX]

**Appendix 1: List of Participants for Semi-Structured Interviews**

|  | **Organization** | **Number of Interview** | **Type of Stakeholders** | **Location** |
| --- | --- | --- | --- | --- |
| **I** | **Key Informant Interviews (33)** | | | |
| 1 | Department of Preventive Medicine, Ministry of Health | 3 (1 male and 2 females) | Policy-Makers | Phnom Penh |
| 2 | Department of Hospital Services, Ministry of Health | 1 (male) | Policy-Makers | Phnom Penh |
| 3 | Department of Planning and Health Information, Ministry of Health | 1 (male) | Policy-Makers | Phnom Penh |
| 4 | Department of Human Resources | 1 (female) | Policy-Makers | Phnom Penh |
| 5 | National Center for Health Promotion | 1 (female) | Policy-Makers | Phnom Penh |
| 6 | Payment Certification Agency | 1 (female) | Policy-Makers | Phnom Penh |
| 7 | National Social Security Fund | 1 (male) | Social Protection Agency | Phnom Penh |
| 8 | MoPoTsyo Peer Education Network | 1 (male) | NGOs (implementer) | Phnom Penh |
| 9 | World Health Organization, Cambodia Office | 1 (male) | NGOs (technical and financial partner) | Phnom Penh |
| 10 | Louvain Cooperation, Cambodia office | 1 (male) | NGOs (technical partner) | Phnom Penh |
| 11 | World Bank, Cambodia Office | 1 (male) | NGOs (technical and financial partner) | Phnom Penh |
| 12 | GIZ (Deutsche Gesellschaft für Internationale Zusammenarbeit), Cambodia office | 1 (male) | NGOs (technical and financial partner) | Phnom Penh |
| 13 | University Research Co., LLC, Health and Social Development Project | 1 (male) | NGOs (technical and financial partner) | Phnom Penh |
| 14 | University of Health Sciences | 1 (male) | Academic | Phnom Penh |
| 15 | Sihanouk Hospital, Center of Hope | 1 (female) | National Hospital | Phnom Penh |
| 16 | Preah Kossamak Hospital | 1 (male) | National Hospital | Phnom Penh |
| 17 | Representative of the Operational District | 5 (1 female, 4 males) | Local Implementers | Siem Reap, Takeo, Kompong Speu, Oddormeanchey, and Prey Veng |
| 18 | Representative of the Provincial Health Department | 5 (5 males) | Local Implementers |  |
| 19 | Representative of the Referral Hospital | 5 (5 males) | Local Implementers |  |
| **II** | **Focus Group Discussion (14)** | | | |
| 1 | Community health workers | 5 groups | Local Implementers | Siem Reap, Takeo, Kompong Speu, Oddormeanchey, and Prey Veng |
| 2 | Healthcare staff | 5 groups | Local Implementers |  |
| 3 | Patients living with T2D and/or HTN | 4 groups | Users and Patient Groups | Siem Reap, Takeo, and Prey Veng |

Abbreviations: NGOs = Non-governmental Organizations; T2D = Type-2 Diabetes; HTN = Hypertension
