# Supplementary material for: Scaling-up integrated type-2 diabetes and hypertension care in Cambodia: what are the barriers to health system performance?
Source: Front Public Health. 2023 Jun 2;11:1136520. doi: 10.3389/fpubh.2023.1136520 (PMC10272385; doi:10.3389/fpubh.2023.1136520)
Supplement: Supplementary file 2 [file Table_2.DOCX]

# **Research Project on Scale-Up of Care for Diabetes and Hypertension in Cambodia**

# **Appendix 2: Interview Guide of Key Informant Interviews**

| **Introduction** |
| --- |
| - **Self-introduction of researcher(s)** - **Background of the research project**   *As you can see, prevalence of people living with type-2 diabetes (T2D) and hypertension (HTN) has increased significantly worldwide as well as in Cambodia. Countries, regardless of income level, including Cambodia are struggling with how to sustainably scale up care of people living with T2D and/or HTN. NIPH has joined an international research consortium (partners from Europe) to conduct a 4-year research project to examine current implementation of care for T2D and HTN in Cambodia and will support the Ministry of Health looking for effective strategies for the scale up of care at public health facilities.*  *We have learnt that you are one of the experts who have work experience related to this topic, and your information will be key to our research. That’s why we would like to have this interview to get your in-depth perspective on* ***the current policy and implementation of care and the scale up****.*   - **Procedure of the interview and consent form**   *The interview will take place for approximately one hour, and this information sheet will give you more information about the research project* [Information sheet handed over]*. As part of ethics requirements, we need your written consent to participate in this interview. This is a consent form. Your participation is totally voluntary and anonymous. Your information will be strictly kept confidential and will be used for the research purpose only.* [Please keep quiet for 1 or 2 minutes allowing the participant to read the consent form]. *Your information is quite important and significant and we are afraid that we cannot note or remember all of them. Therefore, we would like to audio record this interview so that we can transcribe it or listen to it again during the analysis.*  *Do you allow us to record this interview?*  *If you have any question regarding this interview or research project, please feel free to ask us. If you agree and allow us to start the interview now, please sign your name here in this consent form.*  **Outline of interview (topics to cover)—see the attached diagram** |
| **Warm up questions** |
| - Can you briefly describe your position and role within your organization?    - Can you shortly describe the main goals and main activities of your organization? |
| 1. **Care for diabetes and hypertension** |
| Q#1.1 - What is your **understanding** and involvement in the care so far? (what are key elements in your experience?) [knowledge of the current practice and implementation]  Q#1.2 - How do you see the current care for T2D and HTN in prevention and control of NCDs? [Is it enough and would help achieve objectives stated in the policy 2013-2020?-Interest and knowledge]  Q#1.3 – How would you and your organisation see the current implementation of care, taking your organisation’s vision and missions into account? [Would it positively or negatively affect your organisation or your organisation has never discussed it? –Position and Interest]  Q#1.4 – What do you think are major barriers or enablers for the implementation of care? [Knowledge]—[If participants talk about financing, please link it to the financing system immediately.]  Q#1.5 – Can your organisation make any intervention to the current implementation based on what your organisation thinks is right? If yes, can you give us a case your organisation has done? [Leadership and power]  Q#1.6 – Do you know other organisations currently or used to be involved in providing the care? If yes, can you specify them and their involvement? Have you ever worked or collaborated with them? If yes, how do you describe the quality of collaboration? [Alliance] |
| 1. **Policy** |
| Q#2.1 – Are you aware that in the National Strategic Plan for the Prevention and Control of NCDs 2013-2020, there is mention of “provide integrated management of NCDs through primary care” and the development of WHO PEN SOP? [Knowledge]—[Please bring the strategic plan and SOP with you].  Q#2.2 – Is your organisation currently working to achieve the policy or closely implementing the policy? How do you see the realization of the policy? [Interest and position]    Q#2.3 – What do you think are factors (health systems factors or other policies…etc.) supporting or hindering full implementation of this policy? [Knowledge and experience] [If the participants mention financial factor, please link it financing systems immediately.]  Q#2.4 – Does your organisation have resources (human, financial, technological, and others)? [Resources and power]  Q#2.5 – Can your organisation do something to monitor and make this policy better? If no, do you know who or which organisation can do this? [Power and Alliance] |
| 1. **Scale up and recommended strategies** |
| *As our research topic is about scale up (which is also mentioned in the policy), please allow us to get your perspective on the scale up and potential scale up strategies.*  Q#3.1 – Taking the current implementation into account, does your organisation support the scale up? If not, why? [Position and knowledge] [Participants may define the term ‘scale up’ differently. We want to assess their knowledge on this term too.]  Q#3.2 – Here scale up refers to increase in coverage, increase in services available, or explicit mention or change in the policy. Which one do you think is possible, taking the barriers and enablers we have discussed earlier? [Knowledge] [If participants mention financial issues, please link it to financing systems immediately.]  Q#3.3 – Does your organisation have any power or influence in deciding the scale up? If yes, how much (to what extent)? If no, who or which organisations do you think are keys to this? [Leadership and alliance]  Q#3.4 – Who or which organisations or departments do you think might oppose the scale up? How would you describe their power or influence? Why do you think they oppose? [Position and power]  Q#3.5 – According to you, what should we do first for the scale up (any or all the scale up dimensions)—especially if we want to reach vulnerable populations? Would you be able to recommend some scale up strategies? Will your organisation be interested or have some power or resources in carrying out the strategies? |
| 1. **The financing system** |
| Q#4.1 – Do you know any sources of funding for health care, and in particular for “integrated care package” for T2D and HTN in our country?  [Hints for probing: government through taxes, social security/health insurance contribution, out-of-pocket payments by patients/households (through user charge, co-payment…)]  Q#4.2 – If yes, what is the amount of the fund per year and by source?  [Hints: The answer can be in absolute number or relative terms, e.g. %]  Q#4.3 – If yes, how is the fund (mainly government fund) is channeled/disbursed?  [Hints: in the form of supplies of medicines/equipment or transfer cash and how reliable it is]  Q#4.4 – How are health facilities/providers paid (provider payment methods)?  [Hints for probing: fee-for-service, categorical case-based, diagnostic related group, capitation, budget, salary…]  Q#4.5 – Are there any particular arrangements to incentivize providers (financial incentives), in particular providers of “integrated care package” for T2D and HT?  Q#4.6 –How does, in your opinion, the remuneration system of health care provider/health care services, influence the delivery of service for T2D/HTN?  Q#4.7 –What is the payment system for patient to the health services?  [Hints: Out of pocket, Health Equity Fund (HEF), exemption from the hospital, government subsidy, National Social Security Fund (NSSF) and others….]  Q#4.8 –How does, in your opinion, the payment system for patients influence the utilization of health services for NCD?  Q#4.9 – What are the data (cost and patient data) tracking mechanism currently in used? Who are responsible for data handling and how to get access to those data?  [Hints: Available in paper form and data based (PMRS, H2O, other applications….) and probing whether the sources of data have specific allocation for T2D&HT?]  Q#4.10 – What do you think about the current health financing arrangements – what are strengths and weaknesses?  Q#4.11 – Do you have any recommendations to address the weaknesses? |
| **Concluding remark** |
| **-** Do you have any additional remarks?  - Is there something that you think we didn’t cover that is still relevant to this issue/topic?  - Is there someone else you think we should talk to, that you can identify as a key stakeholder? |
