# Supplementary material for: Scaling-up integrated type-2 diabetes and hypertension care in Cambodia: what are the barriers to health system performance?
Source: Front Public Health. 2023 Jun 2;11:1136520. doi: 10.3389/fpubh.2023.1136520 (PMC10272385; doi:10.3389/fpubh.2023.1136520)
Supplement: Supplementary file 3 [file Table_3.DOCX]

**Appendix 3: Focus group guide: outline**

| **Introduction** |
| --- |
| - **Self-introduction of researcher(s)** - **Introduction of SCUBY**   *As you can see, prevalence of people living with type-2 diabetes (T2D) and hypertension (HTN) has increased significantly worldwide as well as in Cambodia. Countries, regardless of income level, including Cambodia are struggling with how to sustainably scale up care of people living with T2D and/or HTN. NIPH has joined an international research consortium (partners from Europe) to conduct a 4-year research project to examine current implementation of care for T2D and HTN in Cambodia and will support the Ministry of Health looking for effective strategies for the scale up of care at public health facilities.*  *Your experience and involvement in receiving/providing/assisting T2D and HTN care are very important and we would like to have a group discussion with all of you to gain your perception, to understand the facilitators, barriers and identify what is the solutions toimprove the current implementation of care and scale-up.*   - **Explain purpose and the intent of the interview:**   *The interview will take place for approximately one hour, and this information sheet will give you more information about the research project* [Information sheet handed over]*. As part of ethics requirements, we need your written consent to participate in this interview. This is a consent form. Your participation is totally voluntary and anonymous. Your information will be strictly kept confidential and will be used for the research purpose only.* [Please keep quiet for 1 or 2 minutes allowing the participants to read the consent form]. *Your information is quite important and significant and we are afraid that we cannot note or remember all of them. Therefore, we would like to audio record this interview so that we can transcribe it or listen to it again during the analysis.*  *Do you allow us to record this interview?*  *If you have any question regarding this interview or research project, please feel free to ask us. If you agree and allow us to start the interview now, please sign your name here in this consent form.* |
| **A. Questions to doctors / health care staff / teams** |
| **CURRENT PROCESS**   1. Can you provide us **details overview of the** **current implementation of care** in your facility? How did the implementation start?  - Which external organizations were involved to support implementation? - Are healthcare workers trained and provided the supporting materials for implementation? (details on training, guidelines and other supporting materials)  1. What are **your experience** (in screening, testing, diagnosis, refer, treatment, health education, follow-up and different level of cooperation)? Probing to:  - Decision on patient’s treatment plan? Patient’s record through paper or database? - Feedback on medication use, treatment, self-management and difficulties patient encountered - Reminder method - Discussion among team at the facility to improve the service and implementation? - How do your healthcare facility reach to the community on sharing knowledge and information?   **FACILITATORS**   1. In your opinion, what do you think are good in the **current implementation of the care** for people with T2D and HTN? 2. What are **important factors that facilitate** the current way of working?  - Human resource, technology, financial arrangement, management etc.   **BARRIERS /OBSTACLES/ FINANCIAL BARRIERS**   1. What are **the obstacles** have you encountered in the care process (screening, testing, diagnosing, retained in care, follow up, self management, different levels cooperation) for patients with T2D/HTN at your facility? 2. What are the reasons for some people who do not come for follow-up regularly? Have you done anything to deal with them? 3. If there is a change in the current process, what do you think are the **obstacles** or still be the problems that hard to change/prevent the change in the process? 4. For the whole current implementation of care, what do you think are posing as major financial barriers to appropriate health care for patients (T2D/HTN/T2D+HTN/T2D with complications/T2D+HTN with complications treatment)?   **SOLUTIONS**   1. From your perspective and experience, what can be done **to improve the current care** of people with T2D and/or HTN? 2. What should be done first in order to extent or scale up the care for T2D and HTN? 3. How do you see the role of community/informal caregiver/ patients as teacher in the process of care? Do you think that these people or other potential people play an important role in care? |
| **B. Questions to Patients** |
| **CURRENT PROCESS**   1. Can you describe how do you first suspect yourself having T2D/HTN?  - Where was the first place you receiving care? Experience at that place? - How did you end up changing to receive treatment from the current healthcare facility? How do you know that the service is available in this facility?  1. What are your **personal experiences** with the **diagnosis/ treatment/health education/self-management and follow up of your disease?** (details on costs, treatment plan, health education received from HCW, practice of self-management and access to equipment for blood pressure or blood glucose check)  - Can you please describe what you do on your own for your health and better management of your disease?  1. Has your **lifestyle changed** since being diagnosed? How? On which items you spend the most money for **your treatment?** Have there been any changes in your health expenditure since you were first diagnosed? What about expenditures on transportation, special food, special health activities? 2. What kind of **home equipment for self care** does your condition require**?** 3. Does your family member or other people (neighbour, friend, volunteer health support group) help you in management of your T2D/HTN or self-management? How? 4. Where are you seeking/gaining the most **knowledge and support** / what kind of self management does it help you/do you use? 5. Are there organisations outside of the healthcare facility that are of help in the management of your disease?   **FACILITATORS**   1. What is good in the **existing process** (about the treatment of your disease, follow up and communication and shared decision making with health care workers, plan care tailored to your needs and circumstances)? 2. What are the important factors that facilitate you in receiving care? Maintain your treatment?   **BARRIERS/FINANCIAL BARRIERS**   1. What do you feel is **not so good in the process?** What can be changed? 2. Are there some **services available which could be useful but unaffordable or difficult to access?** Do you know people who were able to get access to such services? How do they manage? (Do you/ did you face **barriers** to access the services/getting care? Do you know other people who should come to the services, but don’t come? What are the reasons for this?) 3. Where are the **obstacles** that would prevent expansion or change in the process?   **SOLUTIONS**   1. How do you see the **role of patients as teachers/**community/informal caregivers? Who are **potential people/approaches** that can be added to the existing care. 2. In what way do you see the care to develop/change in order to give you the greatest support and to gain the most out of it? 3. Are there any **other financial views (not really barriers)** which could help you to better manage your condition? |
| **C. Questions to community healthworker (Cambodia)** |
| **CURRENT PROCESS:**   - - - 1. What are the reason and process that you are selected to be CHW? What are the benefits for being a CHW?       2. What are your personal experiences as a CHW with people in the community especially people living with T2D/HTN?       3. What are the messages you bring to them? (health education, self-management, reminder etc)   **FACILITATORS**  1. In your opinion, what is good in the **current implementation of the integrated care** for people with T2D and HTN (specify WHO PEN or other relevant program implemented in that site)?  2. What is your **work/communication flow** with the people living in the community and Health Center Management Committee (HCMC)?  3. How well do you **reach and engage** with people in the community?  4. What are important **factors that facilitate** the current way of working?  **BARRIERS/FINANCIAL BARRIERS**  1. What are the **obstacles** in your work as the community health worker? Working flow with community and HCMC?  2. What are the **problems of people who don’t come regularly for care**? **Don’t take action with your message/advise**? What are current actions you take? What are other options?  3. Where are the **obstacles** that would prevent expansion or change in the process?  4. Do you provide **specific services** to the community (especially peer educator such as offer blood glucose/ blood pressure measurement), how is the service charged? How is the **affordability and accessibility** of this services to patients in the community?  5. What according to you posing as **major financial barriers** to appropriate health care for patients with T2D/HTN/T2D+HTN/T2D with complications/ T2D+HTN with complications treatment?  **SOLUTIONS**  1. What are further **options for improving** community engagement in the care of people with T2D and/or HTN?  2. How do you see the **directions of possible extension** of care /scaling up and facilitators in doing that?  3. Who are **potential people/approaches** that can be added to the existing care. How do you see the role of informal caregiver/patients as teacher/neighbour? |
| **Concluding remarks5** |
| **Additional comments**   - Do you have any additional remarks?   **Thank**   - Thank you for your time.   ***Share SCUBY brochure at the end.*** |
